# Ulcérations cutanées révélant une polyangéite microscopique

**DOI:** 10.11604/pamj.2015.22.82.7789

**Published:** 2015-10-01

**Authors:** Naziha Khammassi, Amel Chakroun

**Affiliations:** 1 Service de Médecine Interne, Hôpital Razi, 2010, La Manouba, Tunisie; 2 Faculté de Médecine de Tunis, Service de Cardiologie, Hôpital Universitaire de Bizerte, Tunisie

**Keywords:** ulcération cutanée, vascularite, polyangéite microscopique, skin ulcers, vasculitis, microscopic polyangiitis

## Image en medicine

La polyangéite microscopique (MPA) se définit comme une vascularite nécrosante systémique qui touche les vaisseaux de petite taille, elle appartient à la famille des vascularites à ANCA. L'atteinte rénale et/ ou l'hémorragie alvéolaire représentent le mode de révélation le plus fréquent. Nous rapportons une observation de MPA révélée par des ulcérations cutanées. Patiente de 60 ans était hospitalisée pour prise en charge de plusieurs ulcérations cutanées au niveau des membres inférieurs dans un contexte d'altération progressive de l’état général avec asthénie, myalgies et arthralgies d'allure inflammatoire. L'examen notait à part les lésions cutanées un discret œdème des membres inférieurs mou, blanc gardant le godet. A la biologie il y avait un syndrome inflammatoire biologique (V S à 112 mm H1, CRP à 54mg/l), une anémie normochrome, normocytaire de type inflammatoire (Hb= 10,7 g/dl) et une insuffisance rénale (créatinine à 138 µmol/l). La protéinurie de 24/H était à 0,6g. L’électromyogramme était normal. La radiographie thoracique était sans anomalies. Les étiologies des ulcérations cutanées sont représentées par les causes infectieuses, néoplasiques, dermatologiques et les vascularites. L'examen histologique d'une biopsie cutanée a montré des lésions de vascularite et a permis d’éliminer une cause maligne. Le bilan immunologique (AAN, ANCA) était négatif ainsi que le bilan infectieux. Les anomalies du bilan rénal ont conduit à pratiquer une ponction biopsie rénale. L'histologie révélait une glomérulonéphrite nécrosante extra capillaire segmentaire et focale. Le diagnostic de MPA était retenu et la malade était mise sous corticoïde à la dose de 1mg/kg/j avec une bonne évolution clinique.

**Figure 1:**
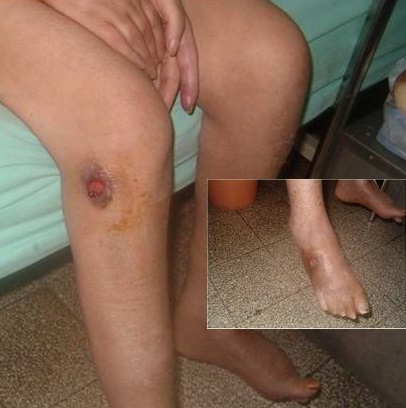
Ulcérations cutanées aux membres inférieurs

